# Digital Transformation of Face-To-Face Focus Group Methodology: Engaging a Globally Dispersed Audience to Manage Institutional Change at the World Health Organization

**DOI:** 10.2196/28911

**Published:** 2022-05-26

**Authors:** Gaya Gamhewage, Mohamed Essam Mahmoud, Anna Tokar, Melissa Attias, Christos Mylonas, Sara Canna, Heini Utunen

**Affiliations:** 1 World Health Organization Genève Switzerland

**Keywords:** qualitative research, digitalization, WHO, World Health Organization, FGDs, focus group discussions

## Abstract

Focus group discussions (FGDs) are widely used to obtain qualitative data from purposely selected groups of people. This paper describes how the Learning and Capacity Development (LCD) unit of the World Health Organization (WHO) Health Emergencies Programme (WHE) digitalized FGDs to engage with WHO staff from around the world, to listen, share, and collect their feedback in the development of a WHO learning framework. The impact of the COVID-19 pandemic and the introduction of local lockdowns and travel restrictions resulted in the wide use of digital platforms, such as Zoom, for employee communications and collaboration capable of reaching employees wherever they are working. The LCD/WHE team drew upon the experience of WHO colleagues from human resources, country, and regional offices to set up and hold FGDs in 6 languages with participants from all WHO regions. Building on the findings of a 2019 WHO staff survey, which was part of a comprehensive, organization-wide career development initiative, the digitalized FGDs allowed for the exchange of substantive feedback, novel ideas, and alignment, connecting across different geographies, disciplines, and levels of seniority. As a result, FGDs can be successfully conducted online, but it is essential to remove barriers to participation by adopting a multilingual and flexible approach in multinational and international organizations such as the WHO.

## Introduction

As part of its transformation process to meet the health challenges of the 21st century, the World Health Organization (WHO) is developing the first-ever global Learning Strategy for health personnel around the world. This Learning Strategy was initiated by the WHO Academy project to provide strategic direction for the operations of the WHO Academy, as well as to frame the broader strategic vision in the domain of learning to achieve health goals that the WHO’s Member States and stakeholders could use as a framework for the future. The WHO Academy is a transformative project to revolutionize lifelong learning in health and is currently being established. Based on this global Learning Strategy, a learning framework is being developed to ensure the upskilling and reskilling of all WHO staff and contractors, establish the norm of lifelong learning, and help transform the WHO into a learning institution. In combination with literature reviews, this process necessitates desk reviews, surveys, and consultations, as well as more in-depth qualitative research on staff views, visions, and suggestions on the “what” and “how” of the learning framework’s role in accompanying them along new career pathways. Learning is in fact one of the key enabling factors that facilitates staff in their career and professional development and constitutes a key requirement for staff to remain up-to-date, relevant, and skilled to perform certain technical roles at their best. The focus group discussions (FGDs) aimed at engaging staff in their career development by identifying the learning needs that they have in relation to their career goals and the challenges they face in acquiring or enhancing certain skills that are critical to advance along a chosen career pathway.

Furthermore, due to the pandemic, it was essential that FGDs be digitized in light of lockdowns and travel restrictions. Therefore, the Learning and Capacity Development unit of the WHO Health Emergencies Programme (WHE) designed a streamlined methodology that ensured staff from all 6 WHO regions and headquarters (HQ) provide their perspectives and perceptions. This paper describes the methodology and the lessons learned from the digitalization of the FGDs to reach WHO staff in all 6 regions and HQ to be used in other contexts where qualitative research is carried out digitally for geographically dispersed populations.

## Methodology

### Designing the FGDs

Based on qualitative research approaches in the health care guidebook [1], a core group of WHE personnel with qualitative research expertise was established to lead the process. The first step was to develop the key principles that would yield the broadest possible participation from a wide range of WHO staff by overcoming already identified barriers. These barriers included geographical dispersion and disengagement from corporate transformation projects; poor internet access; language; and social barriers, such as perceptions of power differentials that may hinder those from the WHO’s country and regional offices from participating; as well as concerns related to the confidentiality and legitimacy of the process itself and its impact toward meaningful change at the WHO. The second step involved scaling up the human resources required to run a large number of FGDs in multiple languages over a limited time frame of 7 days. The third step involved identifying and training a larger research team to ensure that sufficiently robust, high-quality FGDs could be conducted. The final step in the design involved the development of the tools and process for the FGDs, a quality-control mechanism, and a support system for the research team.

### Expanding and Training the Research Team

A total of 27 personnel from the WHE, human resources (HR), and regional offices volunteered to participate as facilitators, notetakers, and hosts for the FGDs. All volunteers were required to attend some 1-hour training sessions to prepare them for their roles. The training aimed to improve facilitation skills focusing on working online and equip them with the necessary digital tools. The facilitators were native or advanced-level Arabic, English, French, Portuguese, Russian, and Spanish speakers.

In order to standardize all sessions, the facilitators were trained to use the FGD script (Multimedia Appendix 1). The FGD script was based on the WHO FGD guidance [2], with questions formulated to reflect the insights gained from a WHO survey conducted in mid-2019 on staff perceptions of career development and learning. The script was tested and then further adapted after holding 2 pilot FGDs in English and French with members of the organizing team. This allowed for collecting feedback from participants and identifying possible bottlenecks including those related to technology failures. In addition, in each FGD session, a notetaker was assigned as “a silent observer” supporting the facilitator by providing notes on various practical aspects that could hinder the smooth running of the FGDs (eg, internet connectivity challenges, which could impact engagement, body language, key messages; Multimedia Appendix 2).

### Inviting and Enrolling the Participants

The research team invited WHO staff to participate in the FGDs using a convenience sampling method [1]; invitations with a complete description of the project were sent via corporate emails with a link to a sign-up form. In addition, as it was essential to create legitimacy, confidence, and trust in the FGDs, the WHO HQ research team collaborated with the corporate HR team to craft the appropriate communication messages to invite staff to participate.

The invitation included a personalized video message by the research team lead outlining the purpose of the survey, highlighting the importance of staff participation, and making a firm commitment by the team to preserving confidentiality and using data appropriately. The combination of formal and personalized invitations by a senior staff member offered increased motivation to participants to enroll in the exercise.

### Conducting the FGD Sessions

All FGDs were conducted through the online videoconferencing platform Zoom. Gender and geographical balance were ensured whenever possible. The sessions started with a plenary meeting where all FGD participants received the same introductions and were then sent into breakout rooms according to their language or group preferences. Standard scripts were used for the plenary and breakout rooms to ensure consistency. The duration of each FGD was approximately 60 minutes. Verbal informed consent was obtained from every participant at the start of the FGDs.

### Processing of FGD Data

The research team video recorded, translated into English, and transcribed all FGDs. All data were uploaded into NVivo (version 12; QSR International). The video recordings were secured for transcription and then deleted for confidentiality reasons, and the identity of the participants was kept anonymous. A full narrative report of the findings was produced. A member with the role of checking, as envisaged by the validation technique used in qualitative research [3], was introduced. The findings were presented at an all-staff seminar to check for accuracy and resonance with staff experiences.

### Ethics Considerations

An ethics review was not applicable for this study because this paper is based on an internal consultation process in the WHO. The consent of all the participants was requested and obtained at the start of each online FGD, and their consent was recorded.

## Results

The participatory approach yielded positive results, with 401 staff enrolling in the study within 5 days, of a total of 8000 WHO staff. Those who signed up to participate were split into groups based on self-identification according to the following criteria:

1. Priority groups (National Professional Officers; women in/seeking a leadership role; young professionals; and general service staff in secretarial, administrative, and logistic functions)
2. Language preference (Arabic, Chinese, English, French, Portuguese, Russian, or Spanish)
3. WHO region (African, Americas, Eastern Mediterranean, European, South-East Asian, Western Pacific, or HQ)
4. Time preference (morning or afternoon Central European Time).

The priority groups were identified from the findings of the 2019 mixed methods survey on WHO staff career development and learning. An individual’s presence in 1 priority group did not preclude them from belonging to others, and indeed, many participants identified themselves as belonging to more than 1 category.

A total of 180 participants were available to participate during the 7 days set for the FGDs. In total, 38 FGD sessions in 7 languages were conducted, with 5 participants on average in each group. Although 45% (180/401) of those who enrolled actually participated, staff from all WHO regions and HQ were represented. In most groups, some participants could not use the video function due to low bandwidth, so observation of body language was limited. However, all were able to use audio.

The findings were arranged as follows:

- General findings across all groups, with the following categories related to learning for staff: expectations, priority transversal skills for all staff, technical or job-specific skills, perceived enablers, perceived disablers
- Specific findings related to learning priorities (general service staff, National Professional Officers, women in/seeking a leadership role, young professionals under 40 years of age)
- Ideas related to the WHO Academy (expectations, priority learning activities, fears)
- Links to the new WHO career pathways initiative (expectations, fears)

To validate the findings, nearly 500 staff from all WHO regions participated in the all-staff seminar where the summary findings were presented. No comments were received that challenged the summary findings. The use of anonymized quotes was described by staff as being powerful. Staff commented that the process of participation in the FGDs was motivating in itself, and for many, this was the first time they felt “heard and seen by colleagues in other parts of the organization,” especially at the global level; it was a learning exercise to hear others’ views and perspectives; it yielded socially positive results (“felt great” or “connected” or “as part of one family”) and they would be happy to participate in future FGDs; it led to an increased willingness to use the methodology online for other purposes; and it resulted in requests to create a forum for the participants to stay connected going forward beyond participating in the FGDs.

The findings were used to revise the first-ever WHO global Learning Strategy, make recommendations for the WHO Academy and the elaboration of learning and career pathways, and develop a learning framework to support staff progress. The methodology will now be used as a standard methodology in the WHE and in other WHO staff engagement initiatives to gain the perspectives of key stakeholders for strategy, program, and policy development in the future. By-products such as an online forum initiated by the participants of the first digital FGDs for staff to stay engaged are also underway.

## Discussion

The WHE designed a qualitative study using online FGDs that ensured staff from all 6 WHO regions and HQ participated to provide their perspectives and perceptions to support establishing the global Learning Strategy for the WHO Academy and to support the elaboration of the learning pathways as a key component of career development. In this paper, we argue that despite the many stated challenges of conducting online qualitative research, FGDs can be successfully conducted online. Many researchers, especially social researchers, faced multiple challenges to continue their face-to-face interactions and fieldwork due to public health security measures imposed by governments worldwide since the start of the COVID-19 pandemic [4]. As a result, a digital and nondigital range of ideas and methods were trialed to continue fieldwork in pandemic times [5]. However, much research was conducted online in previous years, and many examples of online surveys, interviews, and digital ethnographies are available in the literature [6-8].

To successfully digitalize FGDs, it is essential to remove barriers to participation by adopting a multilingual and flexible approach in multinational and international organizations such as the WHO, where staff have busy schedules and are separated geographically and hierarchically. These results are consistent with those of other studies and support the digitalization of interviews and FGDs as the most used qualitative methods [9-11].

Additionally, online FGDs have the potential, when designed with consideration of the organizational and participatory contexts, to yield rich results in the form of eliciting not only knowledge but also sentiment. They have collateral advantages of helping personnel in a dispersed organization to feel more connected with each other and be more seen and heard by the power centers of an organization as well as by peers in other locations, with the positive consequence of generating staff engagement. They offer new means of influencing significant change and strategies of a global organization. These social benefits align with the concept that we are currently experiencing a social age characterized by a less hierarchical structure, participation in problem identification, and cocreation of creative and contextualized solutions, rather than command and control of the power centers of an organization [12].

The research team’s decision to engage all staff allowed us to capture rich and varied ideas, thoughts, opinions, and lived experiences that gave voice to employees’ needs and aspirations across different regions and positions. Moreover, such an approach could contribute to creating trusting relationships and building rapport, and thus could decrease possible information bias. This methodological approach allowed meaningful conversations to take place, recognizing each participant’s active role in the process of knowledge cocreation and, by so doing, increasing equity. We also believe that triangulation could diminish researchers’ bias, which was achieved by collecting data from different facilitators and notetakers. Additionally, we were guided by an emergent research design, which consists in considering the whole process as an iterative cycle in which the preliminary findings of the first FGDs informed the subsequent ones. The key lessons learned can be summarized as follows:

1. Designing the FGDs
  Set the most relevant values and principles as foundations for the design of the online FGDs and explicitly link them to the larger processes of clarifying meaning and significance
  Proactively overcome barriers including physical, social, institutional, and psychological barriers (time zones, language, geographical distance, equity of access in participation, trust, credibility, meaning)
2. Preparing the team
  Train facilitators on competencies for running FGDs and on using the technology
  Do a test run—test methodology and technical tools
  Standardize the tools—formal training and unified scripts
3. Running the FGDs
  Run daily debriefing sessions for the facilitation team and offer facilitator support to answer questions and provide coaching
  Ensure equity by inviting all who signed up—even if there were hundreds, and even if it means innovating and expanding the FGD rollout plan
  Deal with low bandwidth—cameras off when necessary
  Keep to time—do not inconvenience participants
  Plan for the worst-case scenario—have alternate staff available for facilitator, notetaker, and host roles
  Be proactive—send reminders to attendees and staff before the start of the event
  Be flexible—participants and staff may be late, and a group may need to be rescheduled
  Anticipate reductions in turnout, even among confirmed participants, providing an opportunity for them to join another FGD
  Do not assume digital literacy or familiarity with selected tech platforms or tools—the more explanation, the better
  Consider how social cues are different online—the awkwardness of knowing when to speak and difficulty observing body language
  Report back to participants and all other stakeholders while maintaining confidentiality
  Integrate findings concretely into ongoing processes
  Provide results in multiple formats that are targeted to different audiences
4. Expanding the benefits
  Appreciate the facilitation team—provide coaching and certificates
  Use the process to keep personnel engaged in major change initiatives
  Use the methodology beyond research to engage stakeholders, to gain feedback on programs, and in planning
  Capitalize and empower the use of other tools and digital fora to maximize social benefits—a strong sense of community, a sense of contributing to something meaningful, and having a voice

### Conclusions

FGDs can be successfully conducted online. To effectively digitalize FGDs, it is essential to remove barriers to participation by adopting a multilingual and flexible approach. Online FGDs have the potential to yield rich results in the form of eliciting not only knowledge but also sentiment and capturing rich and varied ideas, thoughts, opinions, and lived experiences. This methodological approach allowed for meaningful conversations, recognizing the role of each participant in the process of knowledge cocreation and promotion of equity.

## Appendix

Multimedia Appendix 1 Focus group discussion script for WHO staff learning.

Multimedia Appendix 2 Focus group discussion notes and registration form for WHO staff learning.

## Abbreviations

FGD: focus group discussion
HQ: headquarters
HR: human resources
LCD: Learning and Capacity Development unit
WHE: WHO Health Emergencies Programme
WHO: World Health Organization
