# Supplementary material for: Digital Transformation of Face-To-Face Focus Group Methodology: Engaging a Globally Dispersed Audience to Manage Institutional Change at the World Health Organization
Source: J Med Internet Res. 2022 May 26;24(5):e28911. doi: 10.2196/28911 (PMC9185345; doi:10.2196/28911)
Supplement: Multimedia Appendix 1 [file jmir_v24i5e28911_app1.docx]

### Multimedia Appendix 1. focus group discussion script for WHO staff learning

PLENARY (English)

[Host will open meeting and screen share to display the welcome slide so it is visible as participants join; once the host is set up for recording and breakout rooms, they will notify the plenary facilitator that the facilitator can begin the introduction below]

Hello everyone. My name is ___ and I am a part of the team developing the Learning Strategy for the WHO Academy.

First of all, I would like to thank you for volunteering your time to take part in this important exercise.

The purpose of this meeting is to hear from each of you about how the Academy can provide the best opportunities for you to learn and grow as members of the WHO team. The discussion will build on the feedback we received last year in the WHO staff survey and give us the opportunity to discuss staff learning in more detail.

We have heard and acknowledge how challenging this area has been, which is why we are bringing you all together today. We want to focus on how we can move forward constructively to enable learning and development.

You have been invited to participate because your point of view is very important. All of you have direct experience working for WHO. Your input is very much appreciated and will be used to collect ideas that will shape the development of a staff learning framework.

Please keep in mind that the person who will be moderating your discussion is here as a neutral facilitator. Each focus group will run from the time you move into your breakout rooms until the top of the hour, so your facilitator may sometimes encourage you to move to a new question to keep the discussion on schedule. Please respect the facilitator.

Now, we will be divided into breakout rooms, depending on the language preference you provided, to start the focus group discussion.

[Host will activate breakout rooms and state that he/she is available to troubleshoot any problems; everyone else moves to their breakout rooms]

BREAKOUT ROOMS (by language)

[Facilitator should wait a minute before beginning to give everyone a chance to connect; you can review your expected participant list as a reference]

Hello everyone. My name is ___ and I am here today to help run the focus group discussion with you all. My colleague ___ is here to take notes and assist me.

Before we begin, I would like each of you to turn on your cameras and right-click to select ‘Rename’ so you can ensure your first name or a nickname is your screen name for this discussion. Let’s take a few seconds to do that… [Start screen sharing to display introduction slide during the pause]

I will now explain the methodology of the focus group. I will ask general questions about your learning needs and ambitions to facilitate the discussion. The questions are very general and require no specific knowledge, only your experience and thoughts.

Each of you is free to express your own opinion. You do not have to speak in any particular order, but only one person should speak at a time.

Your views may well differ from others in the group. Remember there are no right or wrong answers.

If there are any questions or discussions that you do not feel comfortable answering or participating in, you should not feel obliged to do so. However, please try to be as involved as possible, and do answer as accurately and honestly as possible.

The discussion will remain confidential and I ask you all to uphold this confidentiality when leaving the room. Your opinions will be transcribed anonymously and will be used by the research team to shape the development of the staff learning framework.

I will record this discussion as it will help me to recall what has been said and prepare a transcript of the session. But, despite being recorded, I would like to assure you that the resulting analysis will contain no information that links your name to specific statements. The video recordings will be secured for transcription and deleted once we are done transcribing.

Do you have any questions so far? Would you like to add any additional rules?

I will now take a minute to go around the room and ask each of you to confirm that you understand this information and agree to participate in the focus group fully under the conditions I described. When I say your name, please unmute yourself and say “I consent” if you agree.

[Stop screen sharing and click button to start recording] OK, let’s begin. To "break the ice", I would first like everyone to introduce themselves. Please give your name, location and your role at WHO. Let’s start with ___ …

Now that we have each introduced ourselves, I'll ask a few questions that I hope each of you will be able to answer and share your thoughts or opinions on…

QUESTIONS

1. To do your current job well in the Organization, what do you need to learn most urgently?

Prompts:

- What technical knowledge and skills do you need to stay/get on top of?
- What transversal competencies do you need? For those of you who are not familiar with this term, transversal skills are those typically considered as not specifically related to a particular job, task, academic discipline or area of knowledge but as skills that can be used in a wide variety of situations and work settings, such as critical thinking, communication and teamwork skills. (UNESCO)

1. We all want to grow, flourish and meet our professional ambitions. Imagine the next position you would like to obtain. What do you need to learn to be ready and able to perform in this next job or role?

Prompts:

- What sort of learning would help you feel ready and well-prepared? Please keep in mind that learning can be formal (through academic institutions), non-formal (through workshops or seminars) and informal (through daily experiences).
- What would motivate you to stay on track and acquire the knowledge/experience you need to move to this new position?

1. [Start sharing screen to display ‘WHO career and learning pathways’ slide] I would like to move the discussion to some big changes at WHO regarding career and learning. Our new proposed career and learning pathway model for WHO staff has four tracks: operations, public health, managerial and leadership. It includes several entry and exit points, and there are “roundabouts” that will allow staff to change tracks during their career within WHO. Learning – the continued acquisition of experience, knowledge and skills – is an essential ingredient to move along this pathway.
   1. In your view, what kind of learning will help you move on this pathway? [Show slide with diagram and then turn off screen sharing]
   2. The WHO Academy will implement the WHO Learning Strategy and deliver staff learning in the future. What is your biggest expectation from the Academy to help you learn and grow?
   3. What is your role in helping other staff learn?

*** If participants are expressing a sense of deep frustration in response to a question, we suggest you as the facilitator take a minute to pause the conversation. Let the participants know that you hear their frustration and ask everyone to describe their biggest frustration point and how we turn that into action. This can be repeated as needed. ***

CONCLUSION (in breakout room)

[Type email address into the chat]

I think this has been a rich discussion. Thank you very much for participating. Your feedback will be extremely valuable as we move forward with crafting the staff learning framework. I hope you have found the discussion as interesting as I have.

If there is anything else you would still like to raise, or if there is anything you are unhappy with or wish to complain about, please contact me later. I wrote our team email in the chat.

I would like to remind you that any comments featured in this report will be anonymous. Once, we finalize the main findings, we will share them with you. Please do keep the contents of today’s discussions to yourself.

Thank you once again. You can now leave the meeting.
