# Supplementary material for: Digital Transformation of Face-To-Face Focus Group Methodology: Engaging a Globally Dispersed Audience to Manage Institutional Change at the World Health Organization
Source: J Med Internet Res. 2022 May 26;24(5):e28911. doi: 10.2196/28911 (PMC9185345; doi:10.2196/28911)
Supplement: Multimedia Appendix 2 [file jmir_v24i5e28911_app2.docx]

### Multimedia appendix 2. Focus group discussion notes and registration form for WHO staff learning

| FGD code: |  | | | | |
| --- | --- | --- | --- | --- | --- |
| Date: |  | | | | |
| FGD starting time: |  | | | | |
| FGD ending time: |  | | | | |
| Facilitator name: |  | | | | |
| Notetaker name: |  | | | | |
| Names of participants | Consent | Age | Gender | Duty station | Email |
|  |  |  |  |  |  |
|  |  |  |  |  |  |
|  |  |  |  |  |  |

Notes (to be filled in by the notetaker)

1. Any important comments or remarks on body language, group dynamics and non-verbal interactions; please also note if connectivity was lost by any participants
2. Main themes and points discussed
3. Question 1: To do your current job well in the Organization, what do you need to learn most urgently?
4. Question 2: What do you need to learn to be ready and able to perform in your next job or role?
5. Question 3: WHO career and learning pathway topics
6. Any potential follow-up questions or changes to the FGD guide
7. Any other comments
